# Diabetes mellitus and symptoms of sleep apnea are associated with adverse delivery outcomes

**DOI:** 10.1186/1758-5996-7-S1-A82

**Published:** 2015-11-11

**Authors:** Letícia Maria Tedesco Silva, Denis Martinez, Cintia Zappe Fiori, Martina Madalena Pedroso, Maria Celeste Osório Wender, Adriani Oliveira Galão, Carolina Caruccio Montanari

**Affiliations:** 1 Hospital de Clínicas de Porto Alegre, Porto Alegre, Brazil

## Background

Diabetes is a well-known obstetric risk factor. Sleep apnea is an emerging risk factor for diabetes and may affect gestation as well. Apnea symptoms such as snoring, tiredness and observed apneas predict the risk for sleep apnea. Hypertension, age and obesity are predictors of apnea, diabetes and gestational complications.

## Objective

To associate risk for apnea detected by questionnaire with delivery complications, controlling for diabetes and classical obstetric risk factors.

## Materials and methods

In a prospective cohort design, 158 women answered a version of the STOP-Bang questionnaire adapted for pregnant women using age cutoff at 35 yrs. and excluding male gender (STOP-Ban); risk for sleep apnea was considered present when score>2. Obstetric history and physical examination were obtained. Both gestational and pre-gestational diabetes were considered for the analyses. The hospital records were reviewed for delivery outcomes. Preterm birth, premature rupture of membranes, non-elective cesarean section, low birth weight, and non-reassuring fetal condition were considered adverse delivery outcomes. Seven classical gestational risk factors were used to adjust the multivariate models.

## Results

Delivery data was obtained from 144 women with a mean (±SD) age of 29±6.6 yrs., gestational age of 24±8.9 weeks; body mass index 28.7±6.5 kg/m^2^, blood pressure 113±16/69±9 mmHg. In this sample, 13 women (8%) had diabetes and 41 (28%) had a positive STOP-Ban. In univariate analysis, diabetes increased 12% (relative risk: 1.12; 95% CI 1.03-1.22) while positive STOP-Ban increased 3.84 (1.48-9.9) times risk of delivery complications (P=0.004). The other significant risks in this sample were previous cesarean section and nulliparity. In multivariate analysis, previous cesarean delivery and nulliparity remained significant. The risk introduced by diabetes increased to 8.7 (1.02-74) while positive STOP-Ban maintained the relative risk at 3.79 (1.33-10.8). The R squared for the model increases from 0.23 to 0.28 when STOP-Ban is included.

## Conclusion

In our study, diabetes in pregnant woman is associated with adverse delivery outcomes, even controlling for classical gestational risk factors. When positive questionnaire for sleep apnea is added to the model, the predictive value increases. Identifying symptoms of sleep apnea in pregnant woman may potentiate the preventive influence of prenatal care in minimizing maternal-fetal morbidity.

